# Evaluation of a Technology-Based Survivor Care Plan for Breast Cancer Survivors: Pre-Post Pilot Study

**DOI:** 10.2196/12090

**Published:** 2019-12-20

**Authors:** Talya Laufer, Bryan Lerner, Anett Petrich, Anna M Quinn, Leah Ernst, Alicin Roop, Janet Knoblauch, Nick C Leasure, Rebecca J Jaslow, Sarah Hegarty, Amy Leader, Andrea Barsevick

**Affiliations:** 1 Division of Population Science Department of Medical Oncology Thomas Jefferson University Philadelphia, PA United States; 2 McGlinn Cancer Institute Reading Health System West Reading, PA United States; 3 Department of Medical Oncology Sidney Kimmel Medical College Thomas Jefferson University Philadelphia, PA United States; 4 Division of Biostatistics Department of Pharmacology and Experimental Therapeutics Thomas Jefferson University Philadelphia, PA United States

**Keywords:** cancer survivor, care plan, technology, patient activation

## Abstract

**Background:**

As of 2016, almost 16 million individuals were cancer survivors, including over 3.5 million survivors of breast cancer. Because cancer survivors are living longer and have unique health care needs, the Institute of Medicine proposed a survivor care plan as a way to alleviate the many medical, emotional, and care coordination problems of survivors.

**Objective:**

This pilot study for breast cancer survivors was undertaken to: (1) examine self-reported changes in knowledge, confidence, and activation from before receipt to after receipt of a survivor care plan; and (2) describe survivor preferences for, and satisfaction with, a technology-based survivor care plan.

**Methods:**

A single group pretest-posttest design was used to study breast cancer survivors in an academic cancer center and a community cancer center during their medical visit after they completed chemotherapy. The intervention was a technology-based survivor care plan. Measures were taken before, immediately after, and 1 month after receipt of the survivor care plan.

**Results:**

A total of 38 breast cancer survivors agreed to participate in the study. Compared to baseline levels before receipt of the survivor care plan, participants reported increased knowledge both immediately after its receipt at the academic center (*P*<.001) and the community center (*P*<.001) as well as one month later at the academic center (*P*=.002) and the community center (*P*<.001). Participants also reported increased confidence immediately following receipt of the survivor care plan at the academic center (*P*=.63) and the community center (*P*=.003) and one month later at both the academic center (*P*=.63) and the community center (*P*<.001). Activation was increased from baseline to post-survivor care plan at both the academic center (*P*=.05) and community center (*P*<.001) as well as from baseline to 1-month follow-up at the academic center (*P*=.56) and the community center (*P*<.001). Overall, community center participants had lower knowledge, confidence, and activation at baseline compared with academic center participants. Overall, 22/38 (58%) participants chose the fully functional electronic survivor care plan. However, 12/23 (52%) in the community center group chose the paper version compared to 4/15 (27%) in the academic center group. Satisfaction with the format (38/38 participants) and the content (37/38 participants) of the survivor care plan was high for both groups.

**Conclusions:**

This study provides evidence that knowledge, confidence, and activation of survivors were associated with implementation of the survivor care plan. This research agrees with previous research showing that cancer survivors found the technology-based survivor care plan to be acceptable. More research is needed to determine the optimal approach to survivor care planning to ensure that all cancer survivors can benefit from it.

## Introduction

As of 2016, almost 16 million individuals were cancer survivors, including over 3.5 million survivors of breast cancer [1]. Because cancer survivors are living longer and have unique health care needs, the Institute of Medicine proposed a survivor care plan to alleviate the many medical, emotional, and care coordination problems of survivors [2,3]. Implementation of the survivor care plan is resource-intensive, requiring time and personnel to create and communicate the plan to survivors and other stakeholders [4,5]. Given the many time demands on health care providers, it is imperative to document the benefits of the survivor care plan to survivors. However, several randomized controlled trials have failed to show the benefits of the survivor care plan in relieving survivor distress, improving satisfaction with care, or improving care coordination [6-8].

A small body of research has not demonstrated the efficacy of the survivor care plan (both paper and electronic documents) in influencing survivor-reported outcomes. Three randomized controlled trials of a survivor care plan paper document and in-person session showed no effect on: cancer-specific or general psychological distress, health-related quality of life, satisfaction, or continuity of care [6], cancer worries, depression, or impact of cancer [7], or the helpfulness of the materials [8]. A technology-generated survivor care plan delivered after surgery and updated during follow-up visits showed a difference in the amount of information received (in favor of the survivor care plan group) but no difference in satisfaction with the information or care [9]. The survivor care plan group also reported more symptoms, expressed more illness concerns, more emotional upset, and reported more contact with their primary care physician.

While it is possible that survivors derive limited benefit from the survivor care plan, an alternative possibility is that other patient-reported outcomes, such as knowledge or confidence, could be better indicators of efficacy. Correlational studies have shown a link between receipt of a survivor care plan and increased knowledge and confidence of survivors [9-12]. Further evidence has been provided by a small randomized trial (N=79) about survivor transition coaching compared to usual care, which showed a trend for higher self-efficacy (an indicator of confidence) in the coaching group [13]. Another small randomized trial comparing two survivor care plan interventions showed increased confidence in both groups [14]. In a single-group study, perceived knowledge increased after the survivor care plan visit [15]. This evidence suggests that knowledge and confidence should be evaluated further as outcomes by which the benefit of a survivor care plan for patients can be measured.

In the context of other chronic diseases, such as diabetes or cardiac disease management, researchers have identified the construct of patient activation, which is defined as the knowledge, skill, and confidence of an individual to manage their disease [16-19]. Based on this construct, Hibbard developed a patient activation measure to document self-efficacy regarding health behaviors, internal health locus of control, and readiness for involvement in care [20,21]. A small study of a technology-based symptom care plan for neurotoxicity during cancer treatment showed a significant improvement in activation from before to after use of the care plan [22,23], suggesting that this outcome could be relevant after cancer treatment. Two other randomized trials showed mixed results: no effect [13] and increased activation after the survivor care plan visit [14]. Because a goal of the survivor care plan is to help survivors to transition from a passive patient role to an active survivor role and become more responsible for their own health care, it is possible that this measure could more precisely document the benefit of survivor care plan delivery.

To address the resource issues associated with the survivor care plan, recent efforts have been made to use technology to automate components of the survivor care plan. These efforts reflect a broader trend to use technology as a means of increasing quality of care and patient outcomes while decreasing burden on health care professionals in both cancer care and health care overall [24-26].

Another line of research has focused on the use of technology-based survivor care plans for personalized survivor care plan delivery. Researchers evaluated a prototype of a smart phone application in a small sample of survivors and providers in a hypothetical situation [27]. Both patients and providers rated the prototype as usable, portable, and accessible. A Web-based program was designed to generate a tailored survivor care plan by incorporating input from the electronic health record and directly from patients [28]. In a sample of 25 breast cancer survivors, self-reported confidence was high before and after receiving the survivor care plan; 70% were very satisfied with it, and usability ratings were high. At least 75% of oncology and primary care providers endorsed the program. Investigators evaluated a technology-based platform with two components, a symptom care plan and a survivor care plan, and found the symptom care component to be feasible, usable, and acceptable to breast cancer patients receiving neurotoxic chemotherapy [22,23]. In contrast, a randomized study comparing two Web-based survivor care plan tools (completed by the provider versus the survivor) showed low completion rates by both groups [29], suggesting that technology alone may not solve the problem of survivor care plan implementation.

In summary, previous research provides some evidence that self-reported survivor knowledge, confidence, and activation may be sensitive to receipt of a survivor care plan, either paper or electronic. However, further research is necessary to examine survivor-reported outcomes and survivor experience associated with use of a technology-based survivor care plan. In addition to evaluating changes in survivor-reported outcomes during the implementation of a technology-based survivor care plan, this research describes preferences for and satisfaction with the technology and format in which the survivor care plan was delivered.

The objectives of this pilot study were to: (1) examine self-reported changes in knowledge, confidence, and activation from before to after receipt of an electronically generated survivor care plan by breast cancer survivors in an academic cancer clinic and a community cancer clinic; and (2) describe survivor preferences for and satisfaction with implementation of an electronic survivor care plan.

## Methods

### Data Collection

The Institutional Review Board approved the research and each participant provided informed consent. Eligible individuals were required to: have pathologically confirmed breast cancer, stages I-III; be over 18 years old; be completing a course of chemotherapy; and be able to understand and read English. Participants recruited from a National Cancer Institute–designated comprehensive academic cancer center and a community-based cancer center were enrolled at the follow-up medical visit after completion of chemotherapy. The study used a single-group pretest-posttest design. Participants completed surveys both before (see Multimedia Appendix 1) and after (see Multimedia Appendix 2) the medical visit and then one month later (see Multimedia Appendix 3) to document changes in knowledge, confidence, and activation as well as preference for, and satisfaction with, the technology-based survivor care plan.

The intervention consisted of a customized survivor care plan that was developed and delivered via the Carevive Survivor Care Planning system, a proprietary cloud-based system. This system uses clinical data input into a proprietary rules engine that automatically generates a draft care plan based on diagnosis, treatment regimen, current clinical practice guidelines, and nationally established quality metrics. The clinician can review, edit, and customize the plan prior to sign off and delivery to the survivor. The customized survivor care plan includes a treatment summary as well as a care plan describing recommended medical tests, appointments to schedule, and links to vetted resources and reading materials about survivor health concerns that are maintained and updated by the vendor. As a survivor’s treatment and disease history progress, providers can input additional information to the planning system and the survivor care plan will be updated accordingly. This feature of the Carevive system decreases the work required of providers in creating survivor care plans. Rather than requiring providers to continuously keep track of recommendations to survivors and update them based on treatment progression, the Carevive system allows them to simply transfer information on treatment and disease progression from the electronic medical record into the Carevive program interface, and then review and sign off on the survivor care plan that is automatically generated. Ideally, the survivor care plan document is delivered to the individual electronically (via email or encrypted flash drive), thus enabling full use of active links to information; however, the survivor care plan document can also be printed and delivered in paper form per the preference of the survivor. The most prominent difference in features between the electronic and paper versions of the survivor care plan is that the electronic version includes embedded links that allow survivors to directly and immediately access educational resources on their personal computers or mobile devices. Within the Carevive platform, data are maintained in a secure database with Health Insurance Portability and Accountability Act–compliant standards of privacy and security.

### Survivor-Reported Outcomes

We evaluated knowledge and confidence with scales created by the researchers to document changes in survivor-reported knowledge of care expectations and confidence about completing the necessary tasks related to the care plan. These scales were used to distinguish knowledge or information deficits and self-efficacy or skill-related gaps. Each scale contained seven items rated on a 4-point scale from strongly disagree to strongly agree. An example of a knowledge item was: “I know which medical tests need to be done over the next year and when to get them done.” A confidence item was: “I am confident that I will get the medical tests done on time over the next year.” Within each scale, items were summed, the total score for each scale ranged from 7 to 28, and higher scores indicated higher knowledge or confidence.

We also measured self-reported survivor activation. The activation survey is a 13-item scale measuring the degree to which individuals feel prepared to actively participate in self-management [20,21]. Items are measured on a 4-point Likert scale with a range of strongly agree to strongly disagree. Total raw scores ranged from 13-52 (lowest to highest activation). Psychometric evaluation in previous research has revealed strong reliability and validity [20]. In groups with chronic illnesses, higher activation scores predicted higher likelihoods of engagement in preventive health behaviors, of seeking out health information, of performing regular self-monitoring at home, and of lower health care utilization [17,19,30]. In addition to being a measurable construct, activation can be influenced by interventions, such as delivery of a survivor care plan, that focus on providing information and resources to guide action [30,31].

### Survivor Preference and Satisfaction

We examined survivor preferences for the fully functional electronic format of the survivor care plan with or without a paper document versus the paper document alone. We asked participants to rate their satisfaction with the chosen format on a 4-point scale from strongly agree to strongly disagree, as well as to rate the acceptability of the content (ie, ease of understanding, helpfulness of the information and resources, satisfaction with the experience, and recommendation to others) on a separate 4-point scale from strongly agree to strongly disagree.

### Statistical Analysis

Demographic characteristics of the subjects were summarized by means and SDs or counts and percentages, as appropriate, stratifying by site (academic or community center). We assessed differences between the sites with two-tailed *t* tests and Fisher’s exact test.

To evaluate the changes in the survivor-reported outcomes, we fit linear mixed models using the MIXED Procedure (PROC MIXED) in SAS 9.4 (SAS Institute Inc, Cary, North Carolina). Fixed effects included site, time point, and a site by time point interaction; a random intercept was allowed for each subject. We considered several possible correlation structures to account for the repeated measures within subject, including unstructured, compound symmetry, and first-order autoregressive, with the final selection, compound symmetry, based on Akaike Information Criterion. Boxplots display the distribution of knowledge, confidence, and activation scores for the subjects at each time point and site. We also used Fisher’s exact test to evaluate the association between preferred survivor care plan format and available demographics characteristics. For all analyses, Cronbach alpha=.05.

## Results

### Demographics

A total of 38 breast cancer survivors agreed to participate in the study, 15 in the academic center and 23 in the community center group (Table 1). The sample was primarily non-Hispanic white and married or partnered, and about half had at least some college education. Overall, 7/15 academic center participants (46%) and 8/23 community center participants (30%) were working at the time of the study.

**Table 1:** Demographic characteristics by clinical setting.

Demographics		Academic Center (n=15)	Community Center (n=23)	*P* value
Age, mean (SD)		57.6 (12.8)	52.3 (12.8)	.22
**Race, n (%)**				**.64**
	African American	4 (27)	3 (13)	
	Caucasian	11 (73)	19 (83)	
	Other	0 (0)	1 (4)	
**Ethnicity, n (%)**				**.14**
	Non-Hispanic	15 (100)	19 (83)	
	Hispanic	0 (0)	4 (17)	
**Marital Status, n (%)**				**.88**
	Single	2 (13)	2 (9)	
	Married or partnered	10 (67)	17 (74)	
	Divorced, separated, widowed	3 (20)	4 (17)	
**Education, n (%)**				**.08**
	High school, vocational/technical	3 (20)	11 (48)	
	College (Associates/Bachelors)	8 (53)	11 (48)	
	Advanced degree	4 (27)	1 (4)	
**Employment, n (%)**				**.76**
	Work full/part-time	7 (47)	8 (35)	
	Unemployed	0 (0)	1 (4)	
	Retired	4 (27)	8 (35)	
	Disabled	4 (27)	4 (17)	
	Homemaker	0 (0)	2 (9)	

### Survivor-Reported Outcomes

**Figure 1:**
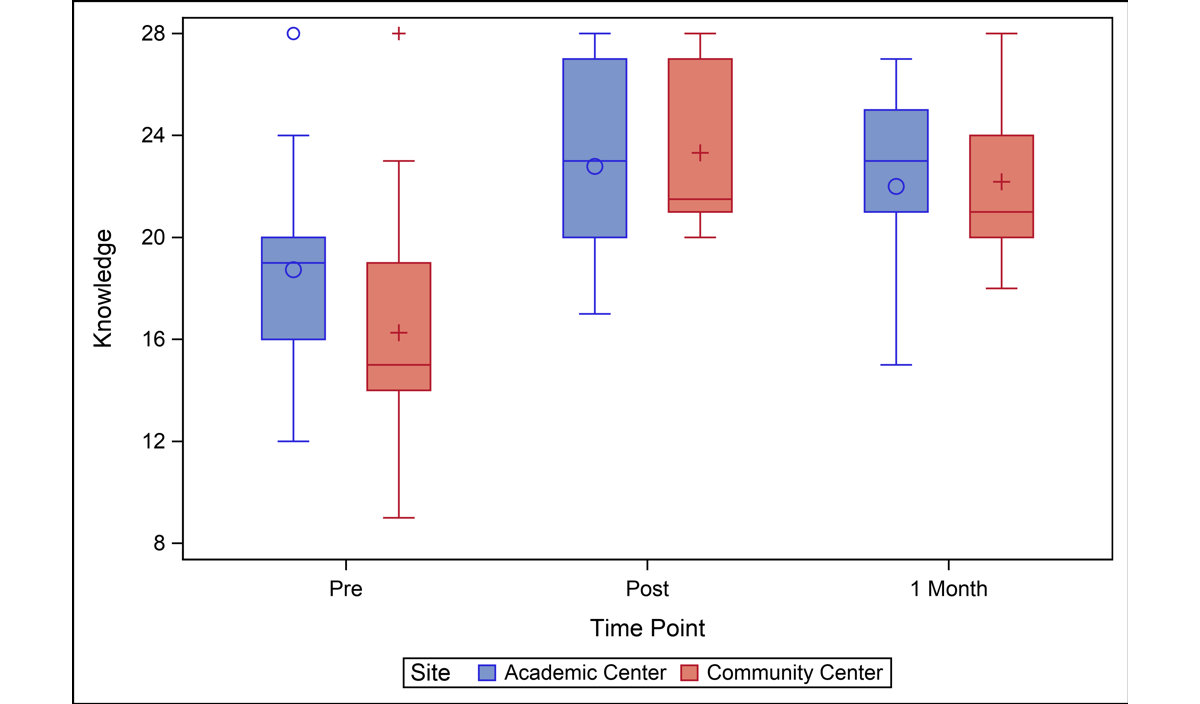
Knowledge levels of study participants before, immediately after, and one-month after SCP presentation. At baseline, the community center group had lower knowledge levels than the academic center group. Knowledge was similar between the sites immediately post-SCP and 1 month later. SCP: survivor care plan.

**Figure 2:**
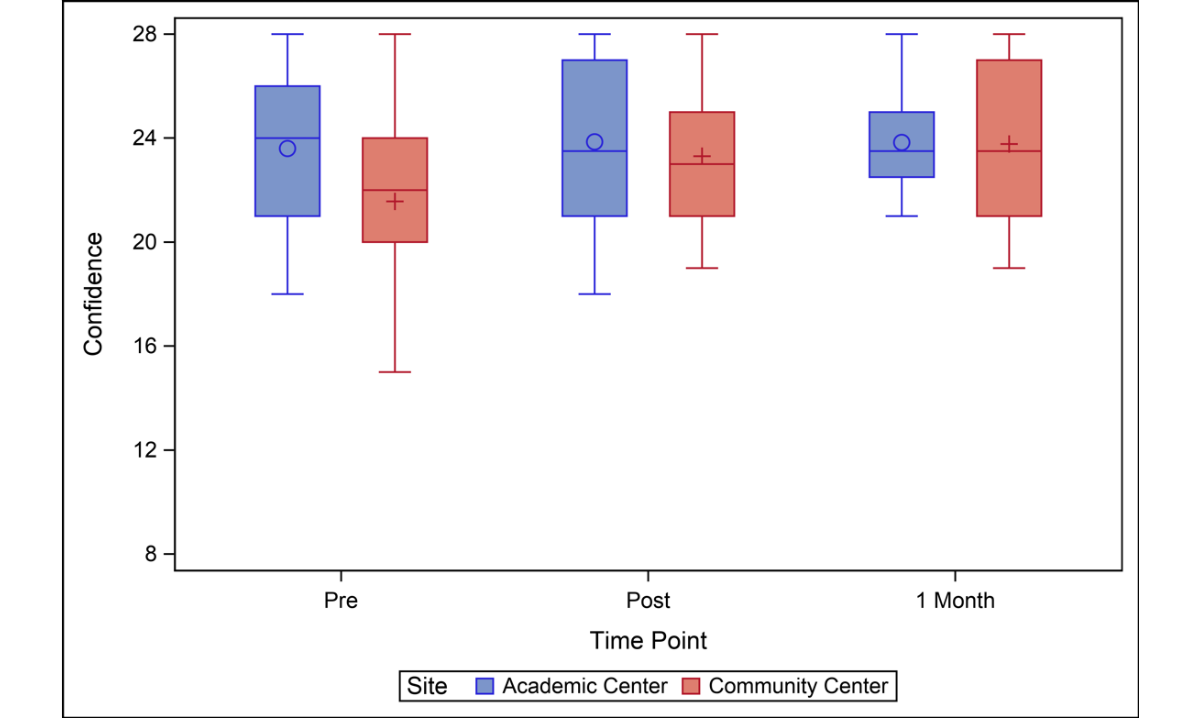
Confidence level before, immediately after, and one-month after SCP presentation. Confidence at baseline was slightly lower in the community center compared to the academic center. Confidence scores of the community center group increased from baseline to immediately and one month post-SCP; confidence levels of the academic center group did not significantly change. SCP: survivor care plan.

**Figure 3:**
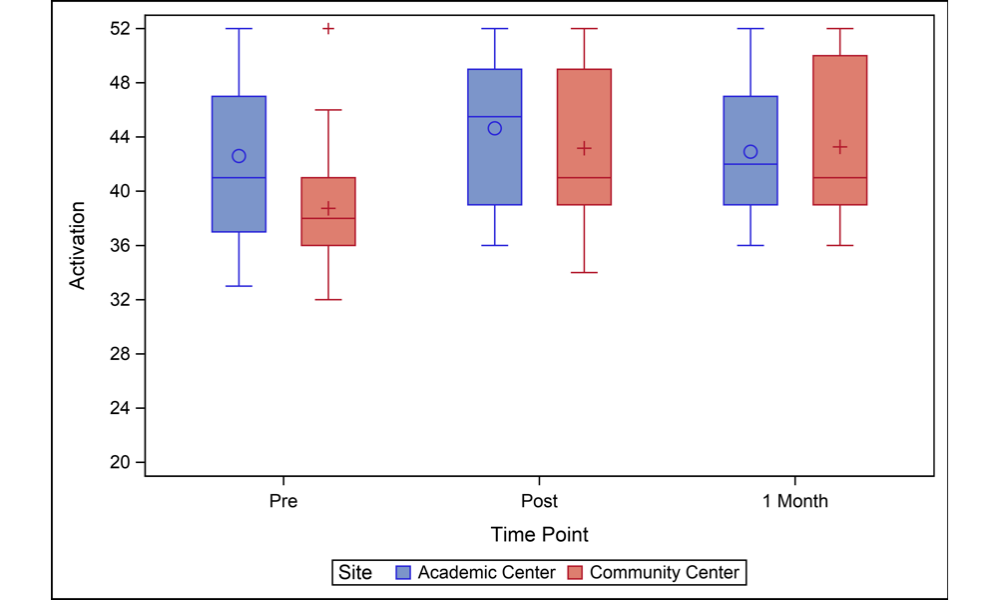
Activation levels before, immediately after, and one month after SCP presentation. Community center participants had lower levels of activation at baseline compared to the academic center. Activation score improved for community center participants at the immediate post and one-month time points relative to baseline. Academic center participants saw modest improvement at the immediately post time point, but the one-month activation scores were not significantly different from baseline. SCP: survivor care plan.

Participants at both sites reported significant increases in knowledge from baseline to immediately after receipt of the survivor care plan and 1 month later (Table 2). At baseline, the community center group had lower knowledge levels than the academic center group (mean difference 2.5; 95% CI 0.0-4.9; *P*=.05). Knowledge was similar between the sites immediately post–survivor care plan and 1 month later (Figure 1). Baseline confidence was also slightly lower in the community center compared to the academic center group (mean difference 2.0; 95% CI 0.1-4.0; *P*=.04) (Table 2). Confidence scores of the community center group increased from baseline to immediately and 1-month post–survivor care plan, and confidence levels of the academic center group did not significantly change (Figure 2). Like knowledge and confidence, community center participants had lower levels of activation at baseline compared to the academic center (mean difference 3.9; 95% CI 0.3-7.5; *P*=.04) (Table 2). The activation score improved for the community center participants at the immediate, post, and 1-month time points relative to baseline. Academic center participants saw modest improvement at the immediately post–survivor care plan time point (change=2.2; 95% CI 0.0-4.5; *P*=.05), but the 1-month activation scores were not significantly different from baseline (Figure 3).

**Table 2:** Model-estimated least squares means for knowledge, confidence, and activation over time for each setting.

Categories			Academic center mean (95% CI)	Community center mean (95% CI)	Difference mean (95% CI)	*P* value
**Knowledge**						
	Pre-SCP^a^		18.7 (16.8-20.7)	16.3 (14.7-17.8)	2.5 (0.0-4.9)	.05
	Post-SCP		22.8 (20.8-24.8)	23.3 (21.7-24.9)	–0.4 (–3.0 to 2.1)	.73
	1 month post-SCP		22.3 (20.3-24.3)	22.1 (20.6-23.7)	0.2 (–2.4 to 2.7)	.91
	**Change from pre to post**		4.1 (2.0-6.2)	7.0 (5.3-8.7)	—^b^	—
		*P* value	<.001	<.001	—	—
	**Change from pre to 1 month**		3.6 (1.4)	5.9 (4.2-7.6)	—	—
		*P* value	.002	<.001	—	—
**Confidence**						
	Pre-SCP		23.6 (22.1-25.1)	21.6 (20.3-22.8)	2.0 (0.1-4.0)	.04
	Post-SCP		24.0 (22.4-25.5)	23.3 (22.1-24.5)	0.7 (–1.3 to 2.6)	.51
	1 month post-SCP		24.0 (22.4-25.6)	23.7 (22.5-24.9)	0.3 (–1.8 to 2.3)	.80
	**Change from pre to post**		0.4 (–1.1 to 1.8)	1.7 (0.6-2.9)	—	—
		*P* value	.63	—	—	—
	**Change from pre to 1 month**		0.4 (–1.1 to 1.9)	2.1 (1.0-3.3)	—	—
		*P* value	.63	—	—	—
**Activation**						
	Pre-SCP		42.6 (39.8-45.4)	38.7 (36.5-41.0)	3.9 (0.3-7.5)	.04
	Post-SCP		44.8 (42.0-47.7)	43.2 (40.9-45.4)	1.6 (–2.0 to 5.3)	.37
	1 month post-SCP		43.3 (40.4-46.2)	43.1 (40.8-45.3)	0.2 (–3.5 to 3.9)	.91
	**Change from pre to post**		2.2 (0.0-4.5)	4.4 (2.7-6.2)	—	—
		*P* value	.05	<.001	—	—
	**Change from pre to 1 month**		0.7 (–1.7 to 3.1)	4.3 (2.5-6.1)	—	—
		*P* value	.56	<.001	—	—

^a^SCP: survivor care plan.

^b^Not applicable.

### Survivor Preference and Satisfaction

Overall, 22/38 (58%) of participants chose the fully functional electronic survivor care plan with or without a paper version (Table 3). It is noteworthy that 12 (52%) of the community center participants requested the paper version of the survivor care plan in comparison to 4 (27%) of the academic center participants. Survivor care plan format preference did not differ between sites with regard to age (*P*=.51), employment status (working versus not working, *P*=.86), or education (high school versus college, *P*=.83) (see Multimedia Appendix 4). All participants were satisfied with their chosen survivor care plan format, and almost all participants at both sites, regardless of chosen format, found the survivor care plan to be easy to understand, with useful information and resources. All but one participant agreed or strongly agreed that the experience was positive and that they would recommend the survivor care plan to other survivors.

**Table 3:** Preference for and acceptability of technology-based survivor care plan by clinical setting.

Categories		Academic center (n=15), n (%)	Community center (n=23), n (%)	*P* value
**SCP format**				**.16**
	Flash drive	3 (20)	1 (4)	
	Paper document	4 (27)	12 (52)	
	Both flash drive and paper document	8 (53)	10 (43)	
**Format useful**				**.74**
	Agree	7 (47)	13 (57)	
	Strongly agree	8 (53)	10 (43)	
**Overall experience**				**.66**
	Negative	1 (7)	0 (0)	
	Positive	3 (20)	5 (22)	
	Very positive	11 (73)	18 (78)	

## Discussion

The results of our examination of the efficacy of the survivor care plan show that participants reported positive changes in knowledge, confidence, and activation from before to after using the technology-based survivor care plan; this suggests that use of the survivor care plan could be responsible for these changes. While the positive change cannot be attributed definitively to the implementation of the survivor care plan, it suggests the need for a randomized controlled trial to test this hypothesis.

We noted that the confidence scores of the community center group increased from baseline to both subsequent time points, while the confidence levels of the academic center group did not significantly change. Community center activation scores were also improved at the immediately post–survivor care plan receipt time point as well as 1 month later, while activation scores at the academic center improved only modestly at the time point immediately post–survivor care plan receipt and at the 1-month time point were not significantly different from baseline. The differences in confidence and activation between the academic and community settings are difficult to explain.

Previous clinical trials evaluating survivor-level outcomes found no effect of survivor care plans on quality of life, mood disturbances, or satisfaction with care [6-8]. This research has identified alternative patient-reported outcomes that may more appropriately capture the variables likely to be influenced by a survivor care plan. Knowledge and confidence have both been identified in many theories as psychological variables that are likely to be involved in behavioral change [32-34]. Activation has also been identified in research on self-management of chronic diseases as an important indicator of readiness to take an active participatory role in one’s health care [17]. Thus, despite a relatively small sample size, the fact that our exploratory study found positive changes in all three of these measures after survivor care plan implementation suggests that survivors may benefit from survivor care plan use, and also invites further investigation of survivor care plan efficacy.

The second goal of this research was to examine uptake of the fully functional electronic version of the survivor care plan versus the paper document only. In this sample, 22/38 (58%) participants chose the electronic survivor care plan, either by itself or in addition to the paper survivor care plan. A total of 34/38 (89%) participants chose the paper format, either by itself or in addition to the electronic survivor care plan. However, only 4/38 (11%) participants elected to receive just the flash drive, compared to 16 (42%) who elected to receive only paper. It could be argued that technology is not fully functional until a substantial majority choose the technology-based option. A platform that functions exclusively electronically thus runs the risk of leaving behind patients who lack the skills to access it and deepening disparities that affect cancer patients and survivors.

Another feature of this study was the comparison between an academic and a community setting. Although we noted differences in survivor care plan format preference by treatment setting, the difference could not be attributed to age, education, or work status differences. However, we did not include any indicators of technological aptitude or savviness that could further explain the difference, such as frequency of interaction with an electronic interface or use of a smartphone. Regarding survivor care plan acceptability, our research agrees with past studies showing that cancer survivors have generally found technology-based survivor care plans acceptable [22,27,28]. Future research should evaluate the influence of technology literacy and aptitude on the choice of survivor care plan format in different clinical settings.

Our study sought to shed light on the potential for this novel electronic platform as a means of generating and delivering survivor care plans to breast cancer survivors. The fact that the survivor care plan examined in our study was offered in two formats strengthened our examination of survivor care plan efficacy by maximizing accessibility to participants. However, because the participants were offered the option of receiving their survivor care plan in both electronic and paper formats it limited our ability to draw conclusions regarding the electronic format, thus ultimately acting as a double-edged sword. Participants who selected both options were not queried about which of the formats they used, and their satisfaction with and perceived benefit from that format.

This pilot study had several additional limitations. First, it was not a randomized controlled trial, so the results are hypothesis-generating and not generalizable. Second, the sample size was small. A third limitation of this research was its lack of focus on clinician perceptions of this technology-based survivor care plan. A more robust evaluation of this intervention would require that all these limitations be addressed.

This research has contributed to the developing body of knowledge about the implementation of technology-based survivor care plans. The identification of patient-reported outcomes that are likely to be influenced by a survivor care plan intervention has been a barrier to success in previous research. It is possible that the outcomes measured in this research (knowledge, confidence, and activation) could be more appropriate indicators of efficacy for paper- or technology-based survivor care plans. Cancer survivors have attested to the acceptability of survivor care plans; however, challenges remain in fully implementing technology-based survivor care plans.

## Appendix

Multimedia Appendix 1 Survey administered to study patients in person before visit with medical oncologist in which SCP was given to them.

Multimedia Appendix 2 Survey administered to study patients in person immediately after SCP was given to them by medical oncologist.

Multimedia Appendix 3 Survey administered to study patients over the phone one month after receiving SCP.

Multimedia Appendix 4 Supplemental Table 1: SCP format selection.
