# Supplementary material for: Evaluation of a Technology-Based Survivor Care Plan for Breast Cancer Survivors: Pre-Post Pilot Study
Source: JMIR Cancer. 2019 Dec 20;5(2):e12090. doi: 10.2196/12090 (PMC6942181; doi:10.2196/12090)
Supplement: Multimedia Appendix 1 [file cancer_v5i2e12090_app1.pdf]

Carevive Pilot Patient Survey Prior To Visit

Patient ID
(TJU_B = 1001-1060, TJU_G = 2001-2060, RH_B = 3001-3060, RH_G = 4001-4060) __________________________________

Name of authorized staff/clinician who administered this survey. __________________________________

Date of survey __________________________________

What is your current marital status?
- ◯ Single
- ◯ Married
- ◯ Partnered/Living together
- ◯ Dating one particular person
- ◯ Separated/Divorced
- ◯ Widowed

Highest school grade completed:
- ◯ 8th grade or less
- ◯ 9 - 11th grade
- ◯ High school graduate / GED
- ◯ Vocational / Technical school
- ◯ Associate degree / some college
- ◯ Bachelor's degree
- ◯ Advanced degree

What is your current employment Status?
- ◯ Employed full-time (more than 32 hrs. per week)
- ◯ Employed part-time (less than 32 hrs. per week)
- ◯ Unemployed
- ◯ Retired
- ◯ On disability/medical leave
- ◯ Homemaker
- ◯ Part-time/Full-time student
- ◯ Working at home
- ◯ Other
- ◯ Response declined

	Strongly disagree	Disagree	Agree	Strongly agree
1. I know which medical tests need to be done over the next year and when to get them done.	◯	◯	◯	◯
2. I am confident that I will get the medical tests done on time over the next year.	◯	◯	◯	◯

	Strongly disagree	Disagree	Agree	Strongly agree
3. I know which medical visits I need to schedule over the next year with my medical providers.	◯	◯	◯	◯

4. I am confident that I will keep these appointments.	○	○	○	○
5. I know what I can do to take an active role in maintaining and improving my health.	○	○	○	○

	Strongly disagree	Disagree	Agree	Strongly agree
6. I am confident that I will take an active role in maintaining and improving my health.	○	○	○	○
7. I know which problems and symptoms to watch for as a cancer survivor.	○	○	○	○

	Strongly disagree	Disagree	Agree	Strongly agree
8. I am confident that I will talk to my medical providers about any problems and symptoms that arise.	○	○	○	○
9. I know what long-term physical effects I may have from cancer and its treatment.	○	○	○	○
10. I am confident that I can cope with the physical effects of cancer and its treatment.	○	○	○	○

	Strongly Disagree	Disgree	Agree	Strongly agree
11. I know what long-term emotional effects I may have from cancer and its treatment.	○	○	○	○
12. I am confident that I can cope with the emotional effects of cancer and its treatment.	○	○	○	○

	Strongly Disagree	Disgree	Agree	Strongly agree
13. I know about my risk for recurrence or new cancers and the risk for my family.	○	○	○	○
14. I am confident that I can deal with the risks for me and my family.	○	○	○	○

	Strongly disagree	Disagree	Agree	Strongly agree
15. When all is said and done, I am the person who is responsible for managing my health.	◯	◯	◯	◯
16. Taking an active role in my own health care is the most important factor in determining my health and ability to function.	◯	◯	◯	◯

	Strongly Disagree	Disagree	Agree	Strongly Agree
17. I am confident that I can take actions that will help prevent or minimize some symptoms or problems associated with my health.	◯	◯	◯	◯
18. I know what each of my prescribed medications do.	◯	◯	◯	◯
19. I am confident that I can tell when I need to get medical care and when I can handle a health problem myself.	◯	◯	◯	◯

	Strongly Disagree	Disagree	Agree	Strongly Agree
20. I am confident that I can tell my health care provider concerns I have even when he or she does not ask.	◯	◯	◯	◯
21. I am confident I can follow through on medical treatment I need to do at home.	◯	◯	◯	◯

	Strongly Disagree	Disagree	Agree	Strongly Agree
22. I understand the nature and causes of my health condition(s).	◯	◯	◯	◯
23. I know the different medical treatment options available for my health condition.	◯	◯	◯	◯

	Strongly disagree	Disagree	Agree	Strongly agree
24. I have been able to maintain the lifestyle changes that I have made for my health.	◯	◯	◯	◯
25. I know how to prevent further problems with my health condition.	◯	◯	◯	◯

	Strongly disagree	Disagree	Agree	Strongly agree
26. I am confident that I can figure out solutions when new situations or problems arise with my health.	○	○	○	○
27. I am confident that I can maintain lifestyle changes like diet and exercise even during times of stress.	○	○	○	○

In the past 3 months did you:

28. Change your diet?
- ○ Yes
- ○ No

If you changed your diet, please select all of the following that apply.
- ☐ To lower calorie intake
- ☐ To increase eating more fruits and vegetables
- ☐ To increase weight
- ☐ To lose weight
- ☐ Other reason

If you selected "Other Reason," please explain.

29. Quit Smoking?
- ○ Yes
- ○ No
- ○ Do not smoke

30. Increase your physical activity or exercise?
- ○ Yes
- ○ No
- ○ Do not exercise

31. Do activities to reduce your stress?
- ○ Yes
- ○ No

If you did activities to reduce stress, please select all of the following that apply.
- ☐ Use mindfulness-based stress reduction (MBSR)
- ☐ Use meditation
- ☐ Do yoga
- ☐ Do another activity to reduce stress

If you do another activity to reduce stress, what is the activity? Please explain.

____

You have now completed all the questions for this portion of the study!

Thank you!
