# Supplementary material for: Evaluation of a Technology-Based Survivor Care Plan for Breast Cancer Survivors: Pre-Post Pilot Study
Source: JMIR Cancer. 2019 Dec 20;5(2):e12090. doi: 10.2196/12090 (PMC6942181; doi:10.2196/12090)
Supplement: Multimedia Appendix 2 [file cancer_v5i2e12090_app2.pdf]

Carevive Pilot Postvisit End Of Visit

Patient ID
(TJU_B = 1001-1060, TJU_G = 2001-2060, RH_B = 3001-3060, RH_G = 4001-4060) ______________________

Name of authorized staff/clinician who administered this survey. ______________________

	Strongly disagree	Disagree	Agree	Strongly agree
1. I know which medical tests need to be done over the next year and when to get them done.	○	○	○	○
2. I am confident that I will get the medical tests done on time over the next year.	○	○	○	○

	Strongly disagree	Disagree	Agree	Strongly agree
3. I know which medical visits I need to schedule over the next year with my medical providers.	○	○	○	○
4. I am confident that I will keep these appointments.	○	○	○	○
5. I know what I can do to take an active role in maintaining and improving my health.	○	○	○	○

	Strongly disagree	Disagree	Agree	Strongly agree
6. I am confident that I will take an active role in maintaining and improving my health.	○	○	○	○
7. I know which problems and symptoms to watch for as a cancer survivor.	○	○	○	○

	Strongly disagree	Disagree	Agree	Strongly agree
8. I am confident that I will talk to my medical providers about any problems and symptoms that arise.	○	○	○	○
9. I know what long-term physical effects I may have from cancer and its treatment.	○	○	○	○

10. I am confident that I can cope with the physical effects of cancer and its treatment.	○	○	○	○

	Strongly Disagree	Disgree	Agree	Strongly agree
11. I know what long-term emotional effects I may have from cancer and its treatment.	○	○	○	○
12. I am confident that I can cope with the emotional effects of cancer and its treatment.	○	○	○	○

	Strongly Disagree	Disgree	Agree	Strongly agree
13. I know about my risk for recurrence or new cancers and the risk for my family.	○	○	○	○
14. I am confident that I can deal with the risks for me and my family.	○	○	○	○

	Strongly disagree	Disagree	Agree	Strongly agree
15. When all is said and done, I am the person who is responsible for managing my health.	○	○	○	○
16. Taking an active role in my own health care is the most important factor in determining my health and ability to function.	○	○	○	○

	Strongly Disagree	Disagree	Agree	Strongly Agree
17. I am confident that I can take actions that will help prevent or minimize some symptoms or problems associated with my health.	○	○	○	○
18. I know what each of my prescribed medications do.	○	○	○	○
19. I am confident that I can tell when I need to get medical care and when I can handle a health problem myself.	○	○	○	○

	Strongly Disagree	Disagree	Agree	Strongly Agree
20. I am confident that I can tell my health care provider concerns I have even when he or she does not ask.	○	○	○	○
21. I am confident I can follow through on medical treatment I need to do at home.	○	○	○	○

	Strongly Disagree	Disagree	Agree	Strongly Agree
22. I understand the nature and causes of my health condition(s).	○	○	○	○
23. I know the different medical treatment options available for my health condition.	○	○	○	○

	Strongly disagree	Disagree	Agree	Strongly agree
24. I have been able to maintain the lifestyle changes that I have made for my health.	○	○	○	○
25. I know how to prevent further problems with my health condition.	○	○	○	○

	Strongly disagree	Disagree	Agree	Strongly agree
26. I am confident that I can figure out solutions when new situations or problems arise with my health.	○	○	○	○
27. I am confident that I can maintain lifestyle changes like diet and exercise even during times of stress.	○	○	○	○

Using Tablet computer:

	Strongly disagree	Disagree	Agree	Strongly agree
28. It was easy to use the tablet computer.	○	○	○	○
29. The screen on the tablet was easy to read.	○	○	○	○
30. I was able to complete the survey quickly.	○	○	○	○

Using Tablet computer:

	Strongly disagree	Disagree	Agree	Strongly agree
31. The questions on the survey were easy to answer.	◯	◯	◯	◯
32. I would recommend the tablet computer survey to others.	◯	◯	◯	◯
33. The treatment summary and survivor care plan I received was easy to understand.	◯	◯	◯	◯

34. I received my treatment summary and survivor care plan in the following format:
(A Flash Drive is a small device with stored info that can be downloaded onto a computer)

◯ Flash Drive ◯ Paper document ◯ Both flash drive and paper document

	Strongly disagree	Disagree	Agree	Strongly agree
35. Getting my treatment summary and survivor care plan in this format was useful.	◯	◯	◯	◯

36. Overall, my experience getting the treatment summary and care plan was:

◯ Very negative ◯ Negative ◯ Positive ◯ Very positive

	Strongly disagree	Disagree	Agree	Strongly agree
37. The survey I completed on the tablet helped me figure out what I wanted from this medical visit.	◯	◯	◯	◯
38. The survey helped me talk with my medical provider about my most important medical concerns.	◯	◯	◯	◯
39. The survey helped me talk with my medical provider about my most important emotional concerns.	◯	◯	◯	◯

	Strongly disagree	Disagree	Agree	Strongly agree
40. The treatment summary and care plan included helpful information to plan my care as a cancer survivor.	○	○	○	○
41. The treatment summary and care plan showed me how to get the resources I need as a survivor.	○	○	○	○
42. I recommend that other cancer survivors receive a treatment summary and care plan.	○	○	○	○

	Very negative	Negative	Positive	Very Positive
43. Overall, I would rate the quality of my medical visit today as.	○	○	○	○

	Strongly disagree	Disagree	Agree	Strongly agree
44. During my medical visit today my provider included me in making decisions about my follow-up care.	○	○	○	○

You have now completed the questions for this portion of the study!

Thank you!
