# Supplementary material for: Evaluation of a Technology-Based Survivor Care Plan for Breast Cancer Survivors: Pre-Post Pilot Study
Source: JMIR Cancer. 2019 Dec 20;5(2):e12090. doi: 10.2196/12090 (PMC6942181; doi:10.2196/12090)
Supplement: Multimedia Appendix 3 [file cancer_v5i2e12090_app3.pdf]

Carevive Pilot 1 Month Follow Up

Patient ID
(TJU_B = 1001-1060, TJU_G = 2001-2060, RH_B = 3001-3060, RH_G = 4001-4060) ______________________

Name of authorized staff/clinician who administered this survey. ______________________

	Strongly disagree	Disagree	Agree	Strongly agree
1. I know which medical tests need to be done over the next year and when to get them done.	○	○	○	○
2. I am confident that I will get the medical tests done on time over the next year.	○	○	○	○

	Strongly disagree	Disagree	Agree	Strongly agree
3. I know which medical visits I need to schedule over the next year with my medical providers.	○	○	○	○
4. I am confident that I will keep these appointments.	○	○	○	○

	Strongly disagree	Disagree	Agree	Strongly agree
5. I know what I can do to take an active role in maintaining and improving my health.	○	○	○	○
6. I am confident that I will take an active role in maintaining and improving my health.	○	○	○	○
7. I know which problems and symptoms to watch for as a cancer survivor.	○	○	○	○

	Strongly disagree	Disagree	Agree	Strongly agree
8. I am confident that I will talk to my medical providers about any problems and symptoms that arise.	○	○	○	○
9. I know what long-term physical effects I may have from cancer and its treatment.	○	○	○	○

10. I am confident that I can cope with the physical effects of cancer and its treatment.	○	○	○	○
	Strongly disagree	Disagree	Agree	Strongly agree
11. I know what long-term emotional effects I may have from cancer and its treatment.	○	○	○	○
12. I am confident that I can cope with the emotional effects of cancer and its treatment.	○	○	○	○
	Strongly disagree	Disagree	Agree	Strongly agree
13. I know about my risk for recurrence or new cancers and the risk for my family.	○	○	○	○
14. I am confident that I can deal with the risks for me and my family.	○	○	○	○
	Strongly disagree	Disagree	Agree	Strongly Agree
15. When all is said and done, I am the person who is responsible for managing my health	○	○	○	○
16. Taking an active role in my own health care is the most important factor in determining my health and ability to function.	○	○	○	○
17. I am confident that I can take actions that will help prevent or minimize some symptoms or problems associated with my health.	○	○	○	○

	Strongly disagree	Disagree	Agree	Strongly agree
18. I know what each of my prescribed medications do.	○	○	○	○
19. I am confident that I can tell when I need to get medical care and when I can handle a health problem myself.	○	○	○	○
20. I am confident that I can tell my health care provider concerns I have even when he or she does not ask.	○	○	○	○

	Strongly disagree	Disagree	Agree	Strongly agree
21. I am confident I can follow through on medical treatment I need to do at home.	○	○	○	○
22. I understand the nature and causes of my health condition(s).	○	○	○	○
23. I know the different medical treatment options available for my health condition.	○	○	○	○

	Strongly disagree	Disagree	Agree	Strongly agree
24. I have been able to maintain the lifestyle changes that I have made for my health..	○	○	○	○
25. I know how to prevent further problems with my health condition.	○	○	○	○

	Strongly disagree	Disagree	Agree	Strongly agree
26. I am confident that I can figure out solutions when new situations or problems arise with my health.	○	○	○	○
27. I am confident that I can maintain lifestyle changes like diet and exercise even during times of stress.	○	○	○	○

In the past month did you:

28. Change your diet?

○ Yes
○ No
(If you checked yes, please respond below)

If you changed your diet, please select all of the following that apply.

☐ To lower calorie intake
☐ To increase fruits and vegetables
☐ To gain weight
☐ To lose weight
☐ For another reason
(If another reason, please respond below)

If you changed your diet for a reason not listed, please explain.

____

29. Quit Smoking?

○ Yes
○ No
○ Never smoked

30. Increase your physical activity or exercise?

○ Yes
○ No

31. Do activities to reduce your stress?

○ Yes
○ No

If you did activities to reduce stress, please select all of the following that apply.

- ☐ Use mindfulness-based stress reduction (MBSR)
- ☐ Use meditation
- ☐ Do yoga
- ☐ Do another activity to reduce stress (if another activity, please respond below)

If you do another activity to reduce stress, what is the activity?

____

Please share with us any thoughts you have about the Survivorship Care Plan you received.

____

You have now completed the questions for each phase of this study!

Thank you for your willingness to support our research!
