# Supplementary material for: Evaluation of a Technology-Based Survivor Care Plan for Breast Cancer Survivors: Pre-Post Pilot Study
Source: JMIR Cancer. 2019 Dec 20;5(2):e12090. doi: 10.2196/12090 (PMC6942181; doi:10.2196/12090)
Supplement: Multimedia Appendix 4 [file cancer_v5i2e12090_app4.pdf]

SCP Format

	Flash Drive Only		Paper document Only		Both flash drive and paper document		*P*
**All**	4	100	16	100	18	100	
**Setting, N (%)**							.16
Jefferson	3	75	4	25	8	44	
Reading	1	25	12	75	10	56	
**Age, mean (SD)**	50.0	7.6	52.8	14.7	56.9	12.1	.51
**Race, N (%)**							.23
Black or African American	2	50	3	19	2	11	
White	2	50	12	75	16	89	
Other	.	.	1	6	.	.	
**Ethnicity, N (%)**							.57
Not Hispanic or Latino	4	100	13	81	17	94	
Hispanic or Latino	.	.	3	19	1	6	
**Marital Status, N (%)**							.83
Single	.	.	1	6	3	17	
Married or partnered	4	100	12	75	11	61	
Divorced, Separated, Widowed	.	.	3	19	4	22	
**Education, N (%)**							.83
HS, Vocational/Technical	1	25	5	31	8	44	
College (AA/BA)	3	75	8	50	8	44	
Advanced degree	.	.	3	19	2	11	
**Employment, N (%)**							.86
Work full/part-time	3	75	6	38	6	33	
Unemployed	.	.	.	.	1	6	
Retired	.	.	6	38	6	33	
Disabled	1	25	3	19	4	22	
Homemaker	.	.	1	6	1	6	
